# Osteopenia Is Associated with Inferior Survival After Deceased-Donor Liver Transplantation

**DOI:** 10.3390/jcm15218659

**Published:** 2026-11-06

**Authors:** Esref Belger, Julian Palzer, Anjali A. Roeth, Clara Antonia Weigle, Bengt Arne Wiemann, Robert Siepmann, Tony Bruns, Verena Keitel-Anselmino, Felix Oldhafer, Oliver Beetz, Thomas Vogel, Florian W. R. Vondran, Iakovos Amygdalos, Franziska A. Meister

**Affiliations:** 1 Department of Surgery and Transplantation, Faculty of Medicine, University Hospital RWTH Aachen, 52074 Aachen, Germanyaroeth@ukaachen.de (A.A.R.); tvogel@ukaachen.de (T.V.); iamygdalos@ukaachen.de (I.A.); 2 Ajmera Transplant Centre, Toronto General Hospital, Toronto, ON M5G 2N2, Canada; 3 Department of Transplantation, Division of General Surgery, University Health Network, Toronto, ON M5G 2C4, Canada; 4 Toronto Centre for Liver Diseases, Toronto General Hospital, Toronto, ON M5G 2C4, Canada; 5 Institute of Radiology, Faculty of Medicine, University Hospital RWTH Aachen, 52074 Aachen, Germany; 6 Department of Internal Medicine III, Faculty of Medicine, University Hospital RWTH Aachen, 52074 Aachen, Germany

**Keywords:** liver transplantation, bone mineral density, osteopenia, body composition

## Abstract

**Background:** Alterations in body composition have been linked to both perioperative and long-term outcomes that cause orthotopic liver transplantation (OLT). Nevertheless, the potential predictive role of recipient bone mineral density (BMD) in clinical outcomes following OLT remains uncertain. In this study, we aimed to explore the role of BMD as a prognostic factor in patients undergoing OLT after brain death donation, in the context of long-term outcome. **Methods:** A total of 480 patients who underwent OLT during the period from 2010 to 2023 at the University Hospital RWTH Aachen were included in the retrospective analysis. Preoperative computed tomography-based segmentation was used to measure BMD at the level of the 11th thoracic vertebra. Osteopenia was defined based previously described sex-specific cutoffs. Perioperative complications were assessed by Clavien–Dindo (CD) classification and comprehensive complication index (CCI). Survival was analyzed using Kaplan–Meier estimates and uni- and multivariable Cox regression. **Results**: In our cohort, osteopenia was highly prevalent (48%). Patients with osteopenia exhibited significantly inferior overall survival (log-rank *p* < 0.001, observed median follow-up 52 months [14–97] vs. 59 months [18–101]) and impaired graft survival (log-rank *p* = 0.037, observed median follow-up 50 months [13–97] vs. 52 months [14–96]). In our multivariable model, osteopenia was confirmed as an independent risk-factor for inferior overall survival (HR 1.602, 95% CI 1.113–2.306; *p* = 0.011) and inferior graft survival (HR 1.379, 95% CI 1.018–1.870; *p* = 0.038). Furthermore, patients suffering from osteopenia required more perioperative red blood cell units (8 [4–13] vs. 6 [3–10], *p* = 0.027). No significant differences in perioperative complications were observed between osteopenic and non-osteopenic patients. **Conclusion**: The presence of osteopenia may represent an easily assessable marker associated with long-term outcome in patients undergoing OLT after brain death donation, thus underlining the predictive significance of body composition in this context. Further research is warranted to validate these findings.

## 1. Introduction

Orthotopic liver transplantation (OLT) is a life-saving intervention for patients suffering from end-stage liver disease, acute liver failure, or hepatocellular carcinoma (HCC) [1,2]. Over the past few decades, there have been remarkable advancements in surgical techniques, immunosuppressive therapies, and post-transplant care that have significantly improved both the perioperative outcome and the overall survival of recipients. Despite these advances, however, perioperative complications remain major challenges that continue to affect patient prognosis [3,4,5]. Recent studies have highlighted the importance of body composition in predicting postoperative outcome in various clinical conditions [6,7,8,9]. Specific components of body composition, including muscle mass, fat tissue, and particularly bone mineral density (BMD), have garnered attention due to their potential to predict both short- and long-term outcomes following oncological surgical procedures. Osteopenia, defined as a pathological decrease in BMD, has been associated with increased perioperative morbidity, an increased incidence of postoperative complications, and inferior overall survival (OS) in various surgical and oncological settings [6,7,8]. Sharshar et al. showed an increased incidence of postoperative pancreatic fistulas after resection of pancreatic carcinomas in patients with lower BMD [6]. Similarly, our group recently demonstrated inferior overall survival in osteopenic patients, after partial hepatectomy for HCC [9]. These findings have led to growing interest in examining whether BMD could serve as a useful predictor of clinical outcomes following OLT. However, the potential predictive role of recipient bone mineral density (BMD) in clinical outcomes following OLT remains uncertain.

Dual-energy X-ray absorptiometry (DXA) is the established reference standard for the assessment of BMD and the diagnosis of osteopenia and osteoporosis. In patients with advanced liver disease, however, DXA is not routinely performed in all transplant candidates, and its accuracy can be impaired by ascites, edema, and degenerative changes in the spine. As an alternative, BMD can be estimated from the attenuation of trabecular bone, measured in Hounsfield units (HU), on CT scans that are routinely acquired during transplant evaluation. CT-derived HU values have been shown to correlate significantly with DXA-derived T-scores [10,11], including in liver transplant recipients [12], and normative values have been established in large cohorts [13]. This opportunistic approach allows the assessment of bone quality without additional examinations, radiation exposure, or costs.

This study aims to evaluate the impact of preoperative BMD, measured using routine computed tomography (CT) scans, on the perioperative and long-term outcome in patients undergoing deceased-donor liver transplantation. By using CT-based segmentation to assess BMD at the level of the 11th thoracic vertebra, we investigate whether BMD could serve as an easily accessible and reliable tool to predict complications and survival in liver transplant recipients. The simplicity of this method, combined with the widespread availability of CT imaging, makes BMD a promising candidate for further evaluation in preoperative risk stratification.

## 2. Material and Methods

### 2.1. Patients and Eligibility

All adult patients (*n* = 496) who underwent a deceased-donor liver transplantation for various indications (see Table 1) at the University Hospital RWTH Aachen, Aachen Germany, between January 2010 and December 2023 were included into this retrospective analysis. Patients with insufficient imaging data (*n* = 16) were excluded from this study. For patients who underwent multiple liver transplantations, only the first one was considered. The institutional review board of RWTH Aachen University (Ethik-Kommission der RWTH Aachen) approved the study (EK 047/18) and waived the requirement for informed consent, as all data were routinely collected in clinical practice and analyzed retrospectively. The study adhered to the principles of the Declaration of Helsinki and ICH-GCP guidelines.

### 2.2. Image Analysis

**Figure 1:**
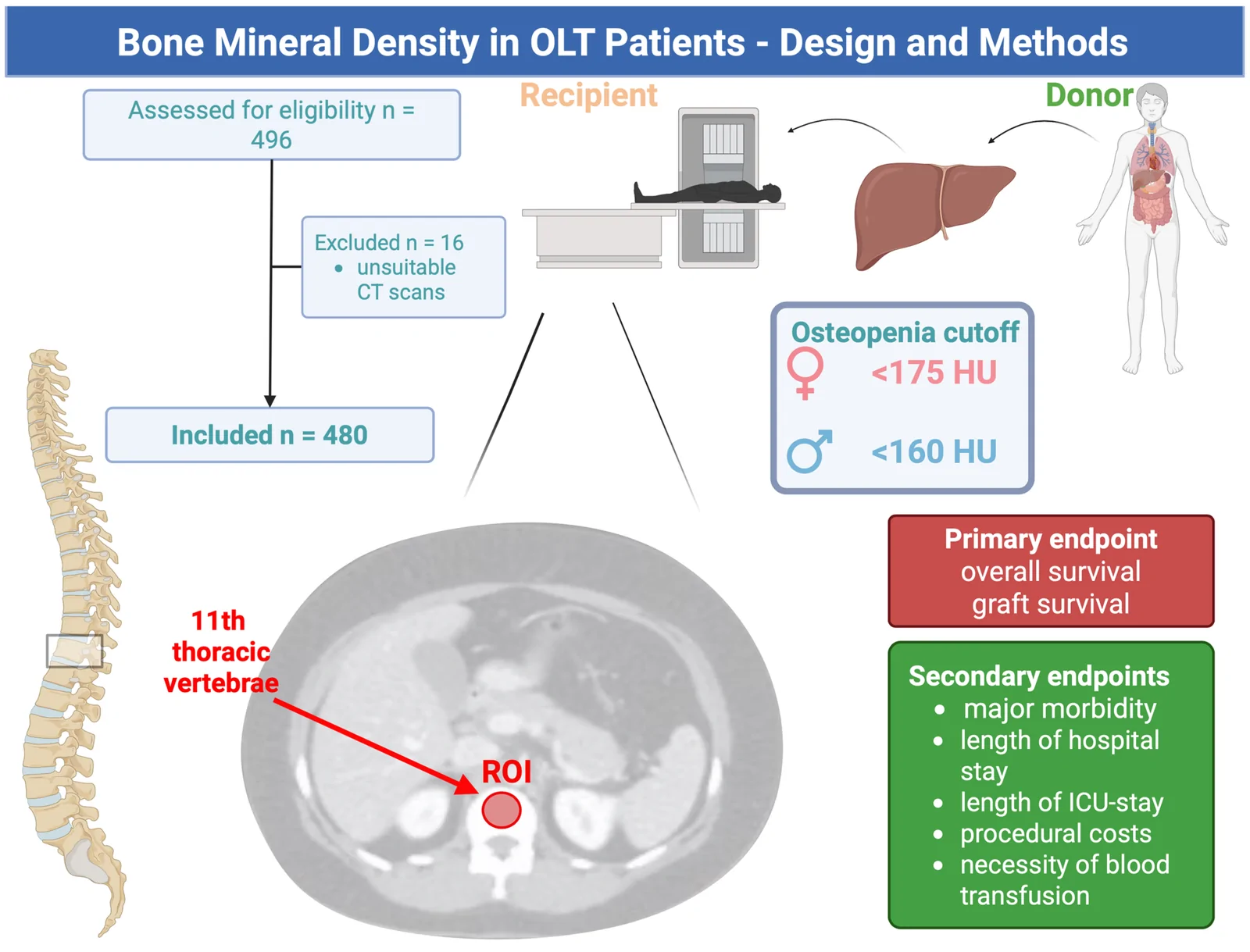
Schematic overview of the study design, bone mineral density assessment, and clinical endpoints. Created in Biorender. Esref Belger. (2024) https://app.biorender.com/illustrations/675648658c9121d58d178ad.

Preoperative CT-based segmentation was used to measure BMD at the level of the 11th thoracic vertebra as described by Sharma et al. [14]. The most recent CT scan prior to OLT was used. Not all patients had a CT scan performed immediately prior to OLT. The preoperative CT imaging used in this study was obtained within a time frame of up to four months before surgery. For all patients, BMD was determined on the non-contrast phase of the CT scans by calculating the mean attenuation within a standardized circular region of interest (ROI), which was placed in the mid-vertebral core and restricted to trabecular bone. The BMD values are specified in Hounsfield units (HU). Osteopenia was defined based on the pre-described and well-established cut-off values of <160 HU for male and <175 HU for female liver transplant recipients [7,9,15] (Figure 1).

### 2.3. Clinical Data Collection

All clinical data were collected in a prospectively maintained institutional database and analyzed retrospectively. Classifications and scores reported in this analysis have been described by our group and by others in previous published studies (including American Society of Anesthesiologists (ASA), Model of End-stage Liver Disease (labMELD), Clavien–Dindo classification (CD) and the Comprehensive Complication Index (CCI) [16,17], procedural costs [18], calculation of transfusion, of the length of hospital stay [2,19,20] and long-term follow-up [21]).

### 2.4. Statistical Analysis

Primary endpoints of this study were overall survival and overall graft survival of patients undergoing a deceased donor liver transplantation. Overall survival was defined as the time from transplantation to death from any cause. Graft survival was defined as the time from transplantation to graft loss, i.e., retransplantation or death (including death with a functioning graft), whichever occurred first. Secondary endpoints were major morbidity defined as CD ≥ 3b, the length of hospital-stay, length of ICU-stay, procedural costs, and the necessity of blood transfusion. Categorical data was reported as absolute and relative frequencies and continuous data were displayed as median (interquartile range-IQR). The Chi-square test and Fisher’s exact test were used to analyze categorical data. Correlations were analyzed using the spearman method. Univariate Cox regression analysis was performed to quantify the effect of the variables on continuous study endpoints. To identify independent risk factors a multivariate Cox regression analysis was performed, by a stepwise forward selection of purposeful selected variables with a *p*-value of <0.200 in the univariate regression analysis and with missing values of <10%. Kaplan—Meier was used to analyze overall survival. Numbers at risk are reported below the survival curves. The interaction between osteopenia and sex was tested by adding a product term to the Cox regression model. Causes of death and of graft loss were extracted retrospectively from the medical records, categorized by the study team and compared between osteopenic and non-osteopenic patients using a permutation chi-square test. Next, OLT recipients have been divided into quartiles, based on the distribution of BMD values over the patient cohort (Q1 ≤ 136.8 HU, Q2 136.8–165.8 HU, Q3 165.8–203.1 HU, Q4 > 203.1 HU; *n* = 120 per quartile). IBM SPSS Statistics version 29.0 (IBM Corp., Armonk, NY, USA) was used to perform all statistical analyses. A *p*-value of <0.05 was considered statistically significant.

**Figure 2:**
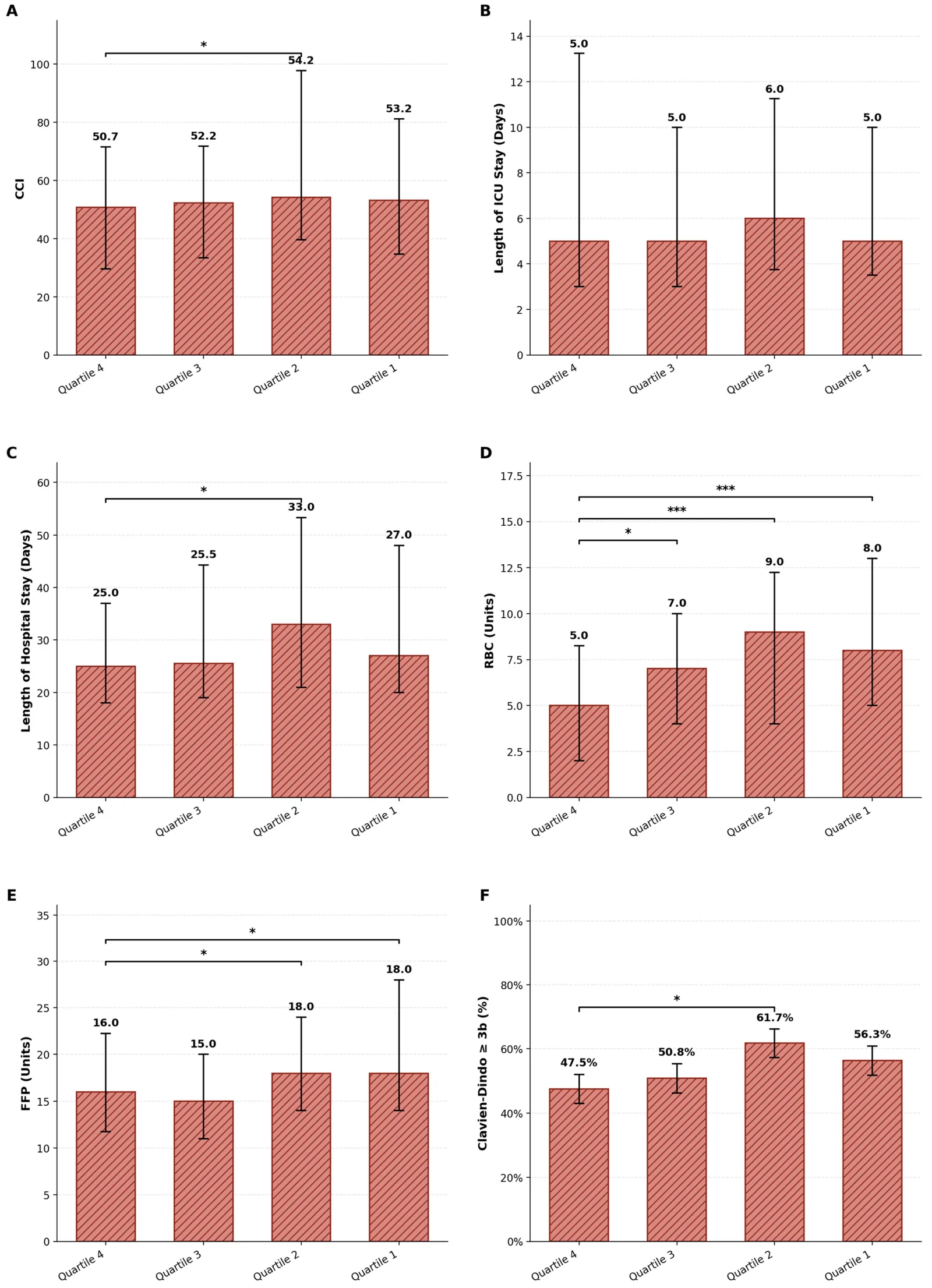
Quartile-based analysis of transfusion requirements, length of hospital stay, length of ICU stay and postoperative complications. Comprehensive Complication Index (CCI) according to BMD (**A**), length of ICU stay according to BMD (**B**), length of hospitalization according to BMD (**C**), intraoperative transfusion of red blood cell (RBC) units according to BMD (**D**), intraoperative transfusion of fresh frozen plasma (FFP) units according to BMD (**E**), Clavien–Dindo (CD) according to BMD (**F**). (median and IQR), * *p* < 0.05, *** *p* < 0.001, one-way ANOVA and Bonferroni post hoc test, *n* = 120, 120, 120, 120, respectively).

**Figure 3:**
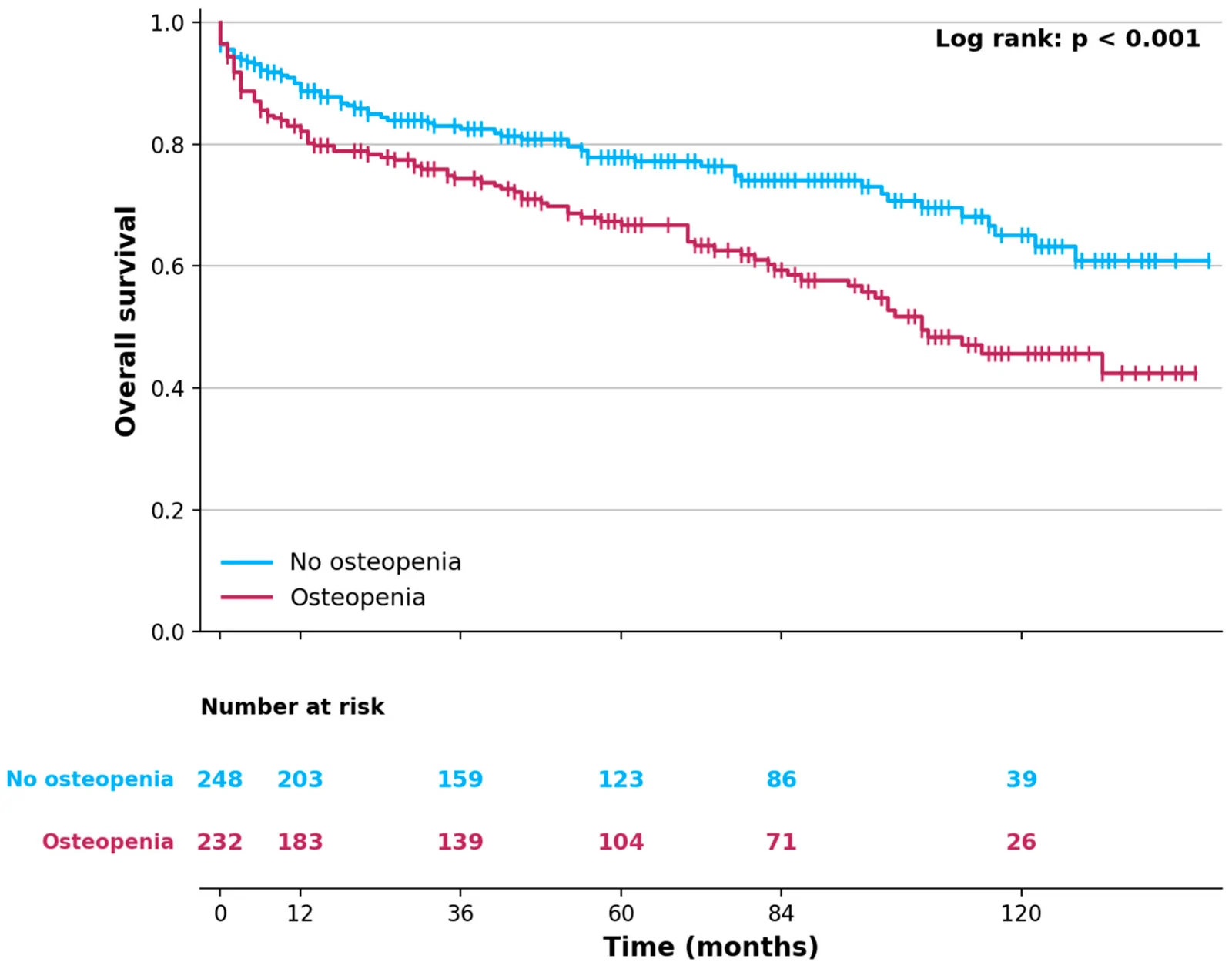
Kaplan–Meier curves for overall survival of patients with and without osteopenia.

**Figure 4:**
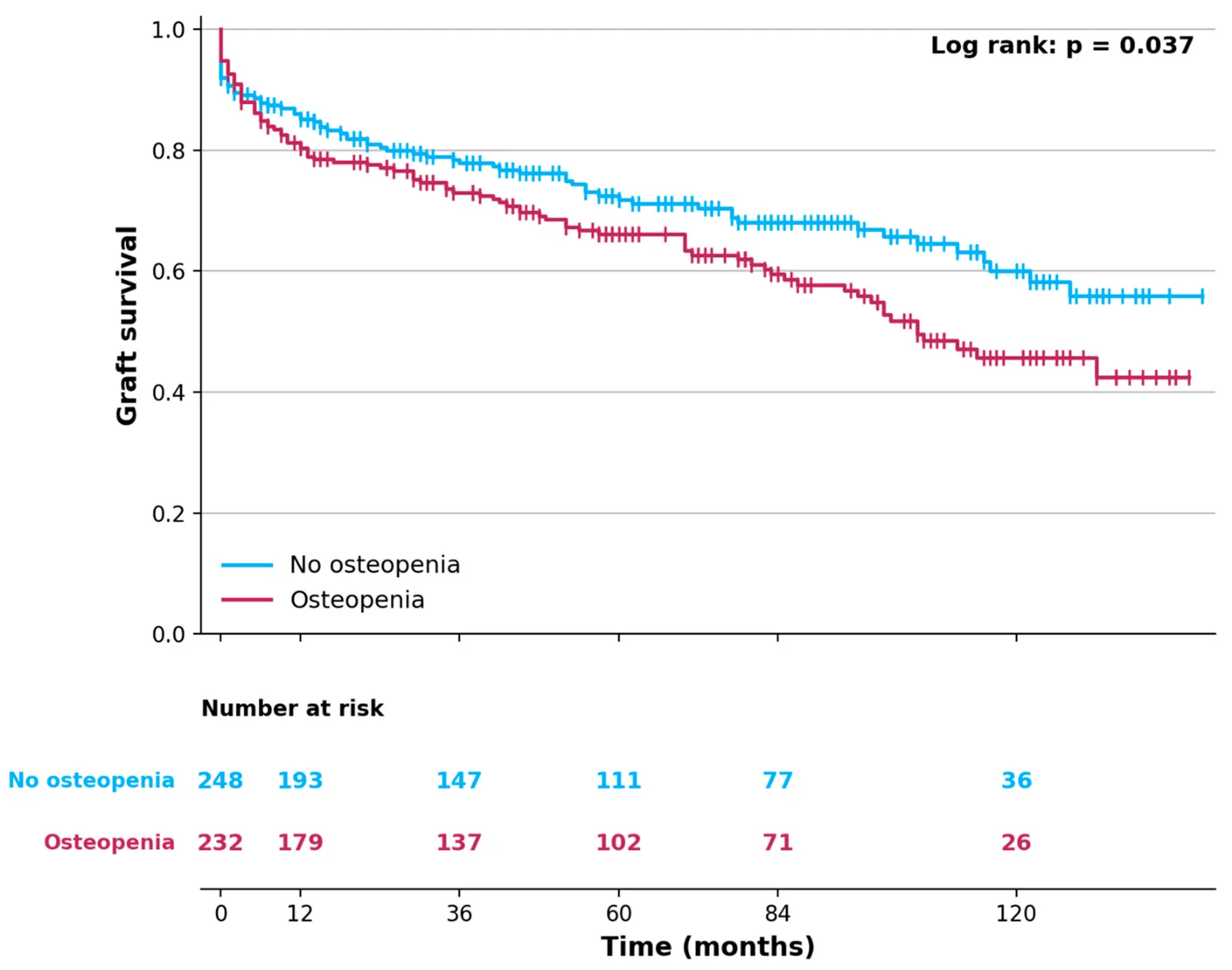
Kaplan–Meier curves for overall graft survival of patients with and without osteopenia.

**Figure 5:**
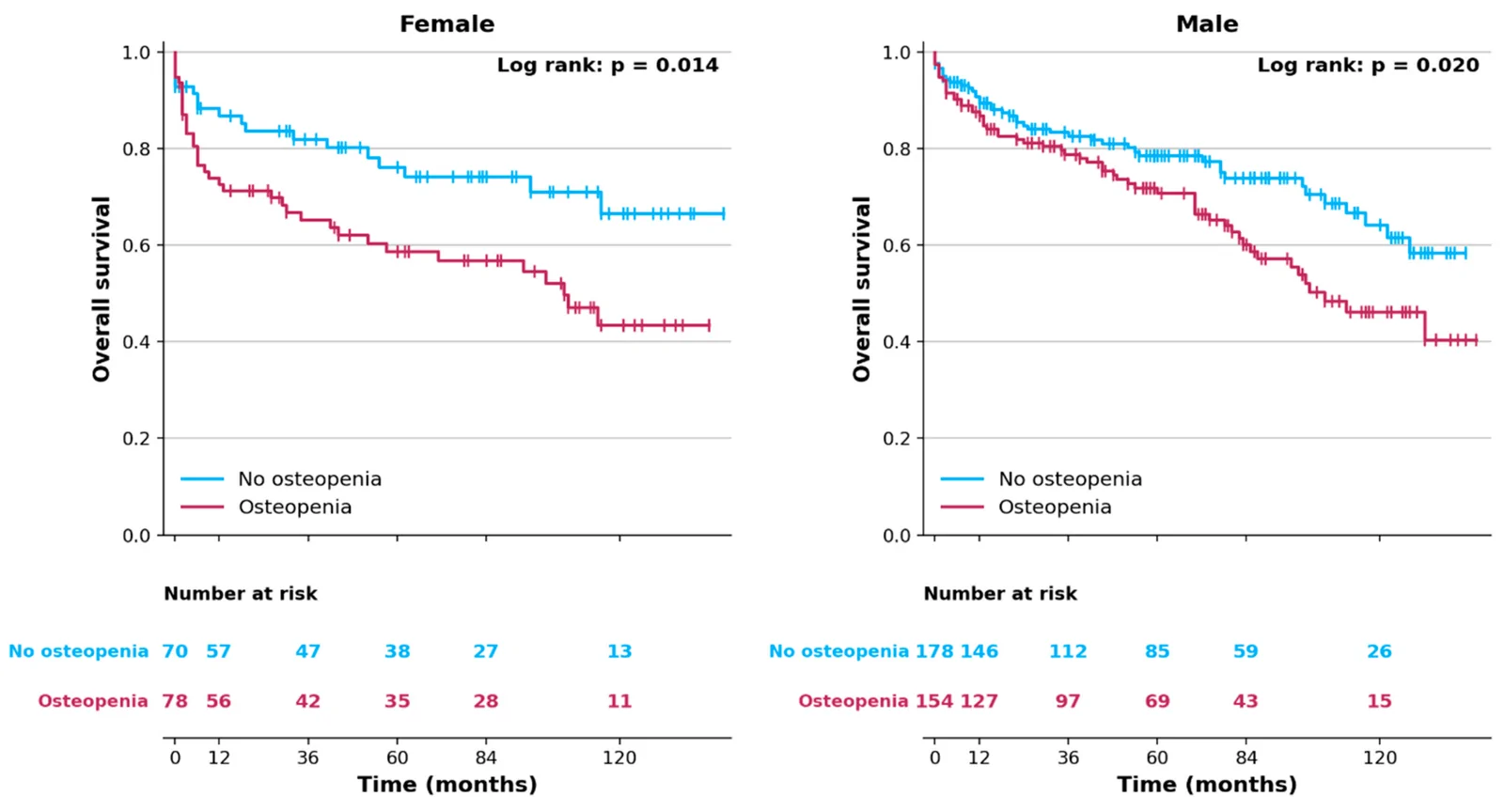
Kaplan–Meier curves for overall survival stratified by sex.

A generative AI tool was used to assist in improving the visual presentation and layout of Figure 2, Figure 3, Figure 4 and Figure 5. The tool was not used for data analysis, interpretation of results, or generation of scientific content.

## 3. Results

### 3.1. Study Population Characteristics

Between January 2010 and December 2023, 496 liver transplantations were performed at the University Hospital RWTH Aachen. Sixteen recipients were excluded due to unsuitable CT scans, resulting in a final study cohort of 480 patients. Of these, 31% were female and 69% male, with a median age of 57 years [IQR 49–63]. The median preoperative lab MELD score was 16 [IQR 10–27]. In accordance with German organ donation legislation, all donors were donors after brain death (DBD), with cerebrovascular accidents representing the predominant cause of death (56%), followed by anoxia (22.5%) and trauma (11%). The indication for liver transplantation differed significantly between patients with and without osteopenia. Patients with osteopenia were significantly less frequently listed for acute liver failure (ALF) compared with those without osteopenia (6.0% vs. 16.1%; *p* < 0.001) (Table 1).

### 3.2. Body Composition Assessment

The median BMD in our cohort was 166 [IQR: 137–203] HU with a median BMI of 27 kg/m^2^ [IQR: 24–30] (Table 1). Taking into account the previously mentioned cut-off values, 248 patients were classified as non-osteopenic and 232 as osteopenic. Age differed significantly between the groups, with osteopenic recipients being older at the time of transplantation than non-osteopenic recipients (53 [IQR: 44–61] vs. 60 [IQR: 54–64] years; *p* < 0.001) (Table 1). However, no statistically significant differences were observed regarding the preoperative labMELD score (15 [IQR: 9–27] vs. 17 [IQR: 10–26]; *p* = 0.322) and the BMI (26 kg/m^2^ [IQR: 23–30] vs. 27 kg/m^2^ [IQR: 24–31]; *p* = 0.622) (Table 1). The median bone density in the non-osteopenic group was 200 [IQR: 179–232] HU, while in osteopenic patients it was 135 [IQR: 115–152] HU (*p* < 0.001, Table 1). Detailed patient characteristics are displayed in Table 1.

### 3.3. Perioperative Outcome and Osteopenia

No significant differences were observed regarding perioperative morbidity (CD ≥3b complications: 50.4% vs. 57.8%, *p* = 0.064), median length of hospital stay (26 [IQR: 19–41] vs. 28 [IQR: 20–51] days, *p* = 0.208), and intensive care stay (5 [IQR: 3–13] vs. 5 [IQR: 3–10] days, *p* = 0.515) between patients without and with osteopenia. In contrast to the findings above, the intraoperative transfusion requirements for RBC were significantly higher in osteopenic patients (RBC: 6 Units [IQR: 3–10] vs. 8 Units [IQR: 4–13]; *p* = 0.027) (Table 2). Nineteen osteopenic patients (8%) and 14 patients (6%) with normal BMD died within the first 90 days after transplantation (*p* = 0.271; Table 2).

In the exploratory quartile-based analysis, isolated differences between individual BMD quartiles were observed, without a consistent gradient across the quartiles. Patients in the lowest BMD quartile (Quartile 1) demonstrated significantly higher Comprehensive Complication Index (CCI) scores compared to Quartile 4 (53.2 vs. 50.7), while the most pronounced difference was observed between Quartile 2 and Quartile 4 (54.2 vs. 50.7; *p* < 0.05). Although median length of ICU stay was comparable across all groups (range 5.0–6.0 days; *p* = 0.3), length of hospitalisation was significantly longer in Quartile 2 compared to Quartile 4 (33.0 vs. 25.0 days; *p* < 0.05). The rate of severe postoperative complications, defined as Clavien–Dindo grade ≥ 3b, was significantly higher in Quartile 2 (61.7%) than in Quartile 4 (47.5%; *p* < 0.05) (Figure 2), whereas the dichotomized comparison between osteopenic and non-osteopenic patients showed no significant difference (Table 2).

### 3.4. Impact of Osteopenia on Long-Term Overall Recipientand Graft Survival

Across the whole cohort, the observed follow-up time had a median of 56 months [IQR: 16–99]. Patients with osteopenia exhibited significantly inferior overall survival (log-rank *p* < 0.001, Figure 3; observed median follow-up 52 months [14–97] vs. 59 months [IQR: 18–101]). Osteopenia was likewise associated with significantly poorer graft survival, in line with the findings for overall survival (log-rank *p* = 0.037, Figure 4, observed median follow-up 50 months [IQR: 13–97] vs. 52 months [IQR: 14–96]).

To identify independent predictors of overall and graft survival, uni- and multivariable Cox regression analyses were performed (Table 3 and Table 4). In univariable analysis, osteopenia was significantly associated with impaired overall survival (HR 1.717, 95% CI 1.246–2.366; *p* < 0.001) and graft survival (HR 1.377, 95% CI 1.016–1.866; *p* = 0.039). Similarly, a preoperative labMELD score ≥16 was associated with both impaired overall survival (HR 2.149, 95% CI 1.401–3.297; *p* < 0.001) and graft survival (HR 1.707, 95% CI 1.162–2.509; *p* = 0.006).

In multivariable analysis, osteopenia remained independently associated with impaired overall survival (HR 1.602, 95% CI 1.113–2.306; *p* = 0.011, Table 3) and graft survival (HR 1.379, 95% CI 1.018–1.870; *p* = 0.038, Table 4). Preoperative labMELD ≥16 was likewise independently associated with both overall survival (HR 1.994, 95% CI 1.140–3.490; *p* = 0.016) and graft survival (HR 1.710, 95% CI 1.164–2.512; *p* = 0.006). None of the other variables included in the respective models showed a significant association with either endpoint.

In the sex-specific analysis, osteopenia was associated with inferior overall survival in both women (log-rank *p* = 0.014) and men (log-rank *p* = 0.020). The association was of similar magnitude in both sexes (women: HR 1.99, 95% CI 1.13–3.51, *p* = 0.017; men: HR 1.58, 95% CI 1.07–2.34, *p* = 0.022). Osteopenic women, however, had a higher early mortality than non-osteopenic women (12-month mortality 27.4% vs. 13.2%; 90-day mortality 12.8% vs. 7.1%), whereas the corresponding differences in men were smaller (13.2% vs. 10.5%; 5.8% vs. 5.1%). Osteopenic women were older than non-osteopenic women (median 59 vs. 49.5 years) and were less frequently transplanted for acute liver failure (10.3% vs. 30.0%). The interaction between osteopenia and sex was not significant (*p* = 0.47; Figure 5).

During follow-up, 156 patients (32.5%) died, 94 of 232 (40.5%) with and 62 of 248 (25.0%) without osteopenia, and 169 patients (35.2%) lost their graft, in 138 of whom graft loss was due to patient death (70.3% of the graft losses in patients without and 90.5% in patients with osteopenia; *p* = 0.001). The most frequent documented cause of death was infection, sepsis or multi-organ failure (71 patients, 45.5%), followed by malignancy (28, 17.9%), cardiovascular events or bleeding (11, 7.1%) and graft failure or other liver-related causes (10, 6.4%); the cause of death was unknown in 26 patients (16.7%) (Table 5). The distribution of causes of death did not differ significantly between osteopenic and non-osteopenic patients (permutation test, *p* = 0.65), although the cause was more frequently unknown in osteopenic patients (20.2% vs. 11.3%). Graft failure not attributable to patient death occurred in 31 patients (9 with and 22 without osteopenia; 3.9% vs. 8.9%; *p* = 0.039), mainly due to vascular complications (*n* = 6), biliary complications (*n* = 6), primary non-function (*n* = 5), recurrent or de novo disease (*n* = 5) and rejection (*n* = 3); the reason was not documented in 5 patients. In an exploratory analysis with death with a functioning graft censored, osteopenia was not associated with a higher risk of graft failure (HR 0.44, 95% CI 0.20–0.95; *p* = 0.035), indicating that the association between osteopenia and impaired graft survival is attributable to patient death rather than to graft failure.

## 4. Discussion

In the present study, we investigated the prognostic relevance of CT-derived BMD in a large cohort of patients undergoing OLT. Osteopenia was highly prevalent, affecting almost half of the study population, and was independently associated with impaired OS and graft survival. Importantly, osteopenia remained independently associated with both endpoints in multivariable Cox regression analysis, alongside the preoperative labMELD score. In addition, patients with osteopenia required significantly more perioperative RBC transfusions, whereas no significant association with postoperative morbidity was observed. Taken together, these findings suggest that CT-derived BMD is associated with long-term outcome independently of established measures of liver disease severity and may serve as an easily accessible marker in liver transplant recipients.

Previous studies have demonstrated an association between reduced BMD and impaired survival in patients undergoing major oncological surgery [6,8,9,14]. In patients with HCC, Miyachi et al. identified osteopenia as a predictor of impaired postoperative survival [7]. Similarly, low BMD has been associated with post-transplant mortality in selected patients undergoing liver transplantation for HCC [14]. Our findings extend these observations to a broader cohort of liver transplant recipients and demonstrate that the prognostic relevance of osteopenia is not restricted to patients undergoing transplantation for malignant liver disease.

Preoperative labMELD ≥ 16 was likewise independently associated with both endpoints, whereas osteopenia retained additional prognostic significance. This finding is of particular interest because labMELD primarily reflects the severity of hepatic dysfunction, whereas reduced BMD may capture additional aspects of physiological reserve, frailty, and cumulative chronic disease burden that are not adequately represented by conventional liver-specific risk scores. The complementary prognostic information provided by these parameters suggests that combining disease-specific scores with CT-derived body-composition markers may allow a more comprehensive characterization of recipient risk.

While the association between reduced BMD and recipient survival is consistent with previous observations, the association with graft survival represents an additional finding of the present study. To our knowledge, the relationship between CT-derived BMD and graft survival following OLT has not been systematically investigated previously. Our findings suggesting an independent association between osteopenia and graft survival indicate that the prognostic value of BMD could potentially extend beyond recipient mortality alone. The mechanisms underlying this association remain uncertain. Reduced BMD may reflect a systemic phenotype characterized by frailty, malnutrition, chronic inflammation, or metabolic dysfunction, which could potentially influence both recipient resilience and long-term graft outcomes. However, the retrospective design of the present study does not permit conclusions regarding causality, and future studies should investigate the relationship between BMD and specific causes of graft loss.

An important methodological aspect is the definition of osteopenia. Although dual-energy X-ray absorptiometry (DXA) remains the clinical reference standard, trabecular bone attenuation can be assessed opportunistically from routinely acquired CT examinations without additional imaging or radiation exposure [6,13]. Previous studies in HCC used a uniform threshold of 160 HU [6]. Given established sex-related differences in bone density [23], we applied previously described sex-specific thresholds of 160 HU for men and 175 HU for women [7,9], comparable to the approach used by Sharshar et al. [6]. In sex-stratified analyses, osteopenia was associated with inferior overall survival in both women and men. No significant differences in the prognostic impact of osteopenia were observed between men and women. This contrasts with previous studies reporting a more pronounced association in male patients [7,9] and may reflect differences in age, underlying disease, cohort composition, and applied thresholds.

The higher prevalence of osteopenia in patients listed for alcoholic-related cirrhosis is biologically plausible and consistent with published data. Chronic liver disease, particularly decompensated cirrhosis and alcohol-related liver disease, is well recognised as a major risk factor for metabolic bone disease. Mechanisms include impaired hepatic hydroxylation of vitamin D, malnutrition, chronic inflammation, hypogonadism, and direct toxic effects of alcohol on osteoblast function. The consequent reduction in bone mineral density observed in patients with alcohol-related liver disease and decompensated cirrhosis has been extensively described in the literature [24,25,26,27,28]. In our cohort, osteopenic patients were significantly more frequently listed for alcoholic-related cirrhosis and significantly less frequently for acute liver failure, suggesting that the osteopenic phenotype may partly reflect the cumulative metabolic burden associated with prolonged chronic liver disease. These aetiology-related differences should be interpreted cautiously.

Interestingly, impaired long-term outcomes in osteopenic patients were not accompanied by significantly increased postoperative morbidity. Previous work from our group demonstrated more major complications in osteopenic patients undergoing liver resection for HCC [9], an association that was not reproduced in the present OLT cohort. This suggests that the adverse long-term outcome associated with osteopenia cannot be explained solely by an increased burden of early postoperative complications.

Nevertheless, osteopenic patients required significantly more perioperative RBC transfusions. In OLT, transfusion requirements are influenced by numerous factors, including portal hypertension, coagulation abnormalities, disease severity, and surgical complexity. Moreover, RBC transfusion itself was not significantly associated with OS or graft survival in our Cox regression analyses. Therefore, a direct causal relationship between reduced BMD, transfusion requirements, and long-term outcome cannot be inferred.

The biological mechanisms linking reduced BMD to adverse outcomes remain incompletely understood. BMD may represent one component of a broader frailty phenotype [29] and is closely related to skeletal muscle status. Sarcopenia has been associated with impaired bone structure [30], while sarcopenia and myosteatosis have themselves been linked to adverse outcomes following OLT [20]. Chronic inflammation, malnutrition, endocrine dysfunction, and altered bone–immune interactions may additionally contribute to both reduced BMD and impaired physiological reserve [31]. Thus, low BMD may be interpreted less as an isolated skeletal abnormality and more as a marker of cumulative systemic disease burden.

From a clinical perspective, CT-derived bone attenuation may therefore complement established risk assessment in liver transplantation. All transplant candidates undergo cross-sectional imaging as part of routine evaluation, allowing BMD to be assessed without additional diagnostic procedures. Combining BMD with other body-composition parameters, such as skeletal muscle mass and myosteatosis, as well as established clinical scores, may provide a more comprehensive assessment of recipient vulnerability [2,3,5,19]. However, whether the addition of BMD improves the predictive performance of existing models requires formal validation.

Assessment of bone health may also have clinical relevance beyond risk stratification. Metabolic bone disease is common in patients with chronic liver disease, and accelerated bone loss and increased fracture risk have been reported particularly during the first year after transplantation [32]. Preoperative identification of reduced BMD may therefore help identify patients requiring further evaluation and preventive measures. Although exercise interventions can improve BMD in other populations [33], the present study cannot establish that increasing BMD would improve recipient or graft survival. Whether interventions targeting bone health, muscle mass, or frailty can modify outcomes following OLT should be addressed prospectively.

Several limitations should be considered. First, the retrospective single-center design limits causal inference and generalizability. BMD is influenced by age, sex, ethnicity, and population-specific factors. Second, CT examinations were performed at varying intervals before transplantation, potentially introducing measurement variability. Third, CT-derived vertebral attenuation is a surrogate of bone density and is not equivalent to a DXA-based diagnosis of osteopenia or osteoporosis. Although DXA remains the clinical reference standard for the diagnosis of osteopenia and osteoporosis, a substantial body of evidence supports the validity of CT-derived vertebral HU measurements as a surrogate for BMD. Several studies have demonstrated significant correlations between trabecular HU values and DXA-derived T-scores across different vertebral levels, with correlation coefficients typically ranging from r = 0.58 to 0.82 [10,11]. Importantly, a study specifically conducted in adult liver transplant recipients showed that CT-derived HU values at the lumbar spine were significantly lower in patients with reduced BMD and demonstrated a graded decline across worsening DXA categories, supporting the feasibility of opportunistic CT-based bone health assessment in this population [12]. Furthermore, Jang et al. established normative trabecular attenuation values for more than 20,000 adults using routine abdominal and thoracic CT [13], providing a robust reference framework for the HU-based approach used in the present study. While CT-derived HU values are not equivalent to a formal DXA-based diagnosis and cannot replace DXA in clinical practice, these data support their use as a valid and practical surrogate in the context of retrospective cohort analyses where DXA data are not routinely available. Finally, residual confounding by factors associated with both reduced BMD and adverse outcome, including frailty, sarcopenia, nutritional impairment, donor factors and disease severity, cannot be excluded. Graft survival was defined as a composite endpoint including death with a functioning graft and therefore largely reflects patient survival; the number of graft failures not attributable to patient death was small (*n* = 31), which precluded a meaningful multivariable analysis of these events. Causes of death and graft loss were extracted retrospectively from clinical records, were not centrally adjudicated and were unknown or not documented in a relevant proportion of deaths (16.7%), more frequently in osteopenic patients. Donor-related variables were not analyzed in this study. Finally, the sex-stratified analyses were exploratory and based on small subgroups; the higher early mortality of osteopenic women, although the interaction was not statistically significant, may indicate residual confounding that we could not assess.

Despite these limitations, our findings demonstrate that reduced CT-derived BMD is independently associated with both overall and graft survival following OLT. Future prospective multicenter studies should validate appropriate BMD thresholds and investigate BMD together with other body-composition parameters. Formal prediction studies are needed to determine whether incorporation of BMD into established clinical models provides incremental prognostic value.

## 5. Conclusions

CT-derived osteopenia is common among patients undergoing OLT and is independently associated with impaired overall and graft survival. Its prognostic relevance despite adjustment for established clinical variables suggests that BMD may capture aspects of recipient vulnerability not fully reflected by conventional measures of liver disease severity. As bone attenuation can be readily assessed from routinely available CT imaging, it may represent a useful additional marker for pre-transplant risk assessment. Prospective studies are required to validate these findings and determine its incremental value within multimodal risk models.

## Figures and Tables

**Table 1:** Patient and procedural characteristics.

Characteristics	All Patients	Osteopenia		*p*-Value
	***n*** ** = 480**	**no ** ***n*** ** = 248**	**yes ** ***n*** ** = 232**	
**Recipient Characteristics**				
**Recipient age (years)**	57 [49–63]	53 [44–61]	60 [54–64]	**<0.001**
**Recipient height (cm)**	174 [167.25–180]	174 [167–178]	175 [168–183]	**0.033**
**Recipient weight (kg)**	82 [70–95]	80 [70–91.38]	83 [70–100]	0.892
**Recipient BMI**	26.5 [23.53–30.18]	26.05 [23.3–29.78]	27.15 [24.1–30.88]	0.622
**Recipient sex ratio (F:M)**	148 (30.8%): 332 (69.2%)	28.2: 71.8	33.6: 66.4	0.201
**Preoperative labMELD**	16 [10–27]	15 [9–27]	17 [10–26]	0.322
**Child–Pugh Score**	7 [5–9]	7 [6–9]	7 [6–9]	0.435
**Preoperative laboratory values**				
**Preoperative Platelets (G/L )**	97 [63–152]	102 [62–164]	95 [63–142]	**0.008**
**Preoperative AST (U/L)**	58 [41–109.75]	63 [41–147]	55.5 [41.25–88.75]	**0.034**
**Preoperative ALT (U/L)**	40.5 [26–78]	45 [28.5–117.5]	37 [23–53.5]	**0.003**
**Preoperative GGT (U/L)**	86.5 [49–203]	101.5 [55–241.75]	78 [43.5–178.25]	**0.026**
**Preoperative Bilirubin (mg/dL)**	2.4 [1.0–8.6]	2.4 [1–12.63]	2.37 [1.025–7.42]	0.314
**Preoperative Albumin (g/dL)**	3.3 [2.8–3.9]	3.4 [2.7–4]	3.3 [2.8–3.7]	0.275
**Body composition**				
**BMD (HU)**	165.75 [136.76–203.07]	200.47 [178.79–231.89]	135.09 [115.2–151.67]	**<0.001**
**Listing indication**				
**ALF**	54 (11.3%)	40 (16.1%)	14 (6.0%)	**<0.001**
**HCC**	141 (29.4%)	67 (27.0%)	74 (31.9%)	0.250
**Alcoholic-related cirrhosis**	92 (19.2%)	37 (14.9%)	55 (23.7%)	**0.011**
**Viral**	29 (6.0%)	20 (8.1%)	9 (3.9%)	0.053
**PSC/PBC**	49 (10.2%)	29 (11.7%)	20 (8.6%)	0.261
**AIH**	9 (1.9%)	3 (1.2%)	6 (2.6%)	0.269
**Other**	106 (22.0%)	52 (21.0%)	54 (23.3%)	0.487

Abbreviations used: BMI: Body mass index; MELD: model for end-stage liver disease; AST: Aspartate aminotransferase; ALT: Alanine aminotransferase; GGT: Gamma glutamyltransferase; BMD (HU); Bone mineral density (Hounsfield units); ALF: Acute liver failure; HCC: Hepatocellular carcinoma; PSC: Primary Sclerosing Cholangitis; PBC: Primary Biliary Cholangitis; AIH: Autoimmune hepatitis.

**Table 2:** Perioperative outcome.

Characteristics	All Patients	Osteopenia		*p*-Value
	*n* = 480	no *n* = 248	yes *n* = 232	
**≥** **CD3b complications**	259 (54%)	125 (50.4%)	134 (57.8%)	0.064
**Hospital stay (days)**	27 [19.25–45.75]	26 [19–41]	28 [20–51]	0.208
**ICU stay (days)**	5 [3–10.75]	5 [3–13]	5 [3–10]	0.515
**Intraoperative RBC transfusion (units)**	7 [4–11]	6 [3–10]	8 [4.25–13]	**0.027**
**Intraoperative FFP transfusion (units)**	16 [13–24]	16 [12–22]	17.5 [14–25.75]	0.054
**CCI**	52.4 [34.6–80.9]	52 [29.9–74.825]	53.450 [36.325–87.375]	0.100
**Lactate ICU admission**	2.85 [1.7–4.53]	3 [1.95–5.3]	2.6 [1.5–4]	**0.020**
**Cost estimation (TEuro)**	52.251 [39.148–78.497]	49.366 [37.854–70.152]	54.906 [41.041–88.000]	**0.023**
**Early allograft dysfunction *****n***** (%)** [22]	109 (22.7%)	58 (23.4%)	51 (22%)	0.325
**90-day mortality ** ***n*** ** (%)**	33 (6.9%)	14 (5.6%)	19 (8.2%)	0.271

Abbreviations used: CD: Clavien–Dindo classification, ICU: Intensive care unit, RBC: Red blood cell units, FFP: Fresh frozen plasma units, CCI: Comprehensive Complication Index, TEuro: Thousand Euros.

**Table 3:** Uni- and multivariable Cox regression analysis for overall survival.

	Univariable Analysis		Multivariable Analysis	
	Hazard-Ratio (95% Confidence Interval)	*p* Value	Hazard-Ratio (95% Confidence Interval)	*p* Value
**Age ≥** 65 years	1.315 (0.865–1.999)	0.199	1.353 (0.820–2.234)	0.237
**BMI ≥ **25	1.038 (0.745–1.446)	0.827		
**Sex** Male	0.858 (0.617–1.194)	0.364		
**Preoperative labMELD ≥** 16	2.149 (1.401–3.297)	**<0.001**	**1.994 (1.140–3.490)**	**0.016**
**Preoperative AST ≥ **40 **(U/L)**	1.173 (0.770–1.788)	0.458		
**Preoperative ALT ≥ 40 (U/L)**	0.821 (0.595–1.134)	0.231		
**Intraoperative FFP **yes	2.305 (0.323–16.476)	0.405		
**Intraoperative RBC** yes	1.468 (0.648–3.326)	0.357		
**Osteopenia** (BMD) yes	**1.717 (1.246–2.366)**	**<0.001**	**1.602 (1.113–2.306)**	**0.011**
**Child–Pugh score ≥ 7**	1.309 (0.907–1.890)	0.151	0.890 (0.565–1.401)	0.614

Abbreviations used: BMI: Body mass index; MELD: Model for end-stage liver disease; AST: Aspartate aminotransferase; ALT: Alanine aminotransferase; FFP: Fresh frozen plasma; RBC: Red blood cell unit; BMD: Bone mineral density.

**Table 4:** Uni- and multivariable Cox regression analysis for graft survival.

	Univariable Analysis		Multivariable Analysis	
	Hazard-Ratio (95% Confidence Interval)	*p* Value	Hazard-Ratio (95% Confidence Interval)	*p* Value
**Age ≥** 65 years	1.198 (0.796–1.803)	0.386		
**BMI ≥** 25	0.906 (0.662–1.239)	0.536		
**Sex** Male	0.841 (0.612–1.154)	0.283		
**Preoperative labMELD ≥ **16	**1.707 (1.162–2.509)**	**0.006**	**1.710 (1.164–2.512)**	**0.006**
**Preoperative AST ≥ **40 **(U/l)**	1.075 (0.724–1.598)	0.720		
**Preoperative ALT ≥ 40 (U/l)**	0.821 (0.602–1.120)	0.214		
**Intraoperative FFP **yes	1.030 (0.255–4.154)	0.967		
**Intraoperative RBC **yes	1.440 (0.675–3.073)	0.346		
**Osteopenia** (BMD) yes	**1.377 (1.016–1.866)**	**0.039**	**1.379 (1.018–1.870)**	**0.038**
**Child–Pugh score ≥ 7**	1.068 (0.757–1.506)	0.710		

Abbreviations used: BMI: Body mass index; MELD: model for end-stage liver disease; AST: Aspartate aminotransferase; ALT: Alanine aminotransferase; GGT: Gamma glutamyltransferase; FFP: Fresh frozen plasma; RBC: Red blood cell unit; CD: Clavien–Dindo classification; BMD: Bone mineral density.

**Table 5:** Causes of death and graft loss in patients with and without osteopenia.

	All Patients	No Osteopenia	Osteopenia	*p*
**Causes of death, ** ***n***	**156**	**62**	**94**	**0.65 (a)**
Infection/sepsis/MOF	71 (45.5%)	27 (43.5%)	44 (46.8%)	0.74
Malignancy	28 (17.9%)	13 (21.0%)	15 (16.0%)	0.52
Cardiovascular/bleeding	11 (7.1%)	5 (8.1%)	6 (6.4%)	0.75
Graft failure/liver-related	10 (6.4%)	6 (9.7%)	4 (4.3%)	0.20
Neurological	7 (4.5%)	3 (4.8%)	4 (4.3%)	1.00
Other	3 (1.9%)	1 (1.6%)	2 (2.1%)	1.00
Unknown/not documented	26 (16.7%)	7 (11.3%)	19 (20.2%)	0.19
**Graft losses, ** ***n***	**169**	**74**	**95**	**0.007 (a)**
Due to patient death	138 (81.7%)	52 (70.3%)	86 (90.5%)	0.001 (b)
Graft failure not due to patient death	31 (18.3%)	22 (29.7%)	9 (9.5%)	0.001 (b)
Vascular complications	6 (3.6%)	4 (5.4%)	2 (2.1%)	0.41
Biliary complications/ischemic cholangiopathy	6 (3.6%)	3 (4.1%)	3 (3.2%)	1.00
Primary non-function	5 (3.0%)	4 (5.4%)	1 (1.1%)	0.17
Recurrent or de novo disease	5 (3.0%)	4 (5.4%)	1 (1.1%)	0.17
Rejection	3 (1.8%)	1 (1.4%)	2 (2.1%)	1.00
Infection	1 (0.6%)	1 (1.4%)	0 (0.0%)	0.44
Reason not documented	5 (3.0%)	5 (6.8%)	0 (0.0%)	0.015

Causes were categorized retrospectively from the medical records; percentages refer to the number of deaths or graft losses in the respective column. MOF: multi-organ failure. *p*-values are exploratory and unadjusted for multiple testing: Fisher’s exact test for each row; (a) overall comparison of the distribution of causes of death and graft loss (including patient death) between groups (permutation chi-square); (b) graft loss due to patient death vs. graft failure for other reasons. The overall distribution of reasons for graft failure not due to patient death did not differ between groups (permutation test, *p* = 0.45).

## Data Availability

All relevant data were reported within the manuscript. Further supporting data will be provided upon written request addressed to the corresponding authors.

## Abbreviations

AIH	Autoimmune hepatitis
ALF	Acute liver failure
ALT	Alanine Aminotransferase
ANOVA	Analysis of Variance
ASA	American Society of Anesthesiologists
AST	Aspartate Aminotransferase
BMD	Bone Mineral Density
BMI	Body Mass Index
CCI	Comprehensive Complication Index
CD	Clavien–Dindo classification
CI	Confidence Interval
COD	Cause of death
CT	Computed Tomography
CVA	Cerebrovascular accident
FFP units	Fresh Frozen Plasma units
GCP	Good Clinical Practice
GGT	Gamma-Glutamyl Transferase
HCC	Hepatocellular carcinoma
HU	Hounsfield unit
ICU	Intensive Care Unit
MELD	Model for End-stage Liver Disease
MOF	Multi-organ failure
OLT	Orthotopic liver transplantation
OS	Overall survival
PBC	Primary biliary cholangitis
PSC	Primary sclerosing cholangitis
RBC unit	Red Blood Cell units
ROI	Region of interest
TEuro	Thousand Euros
